# Supplementary material for: Flavonoids in Amomum tsaoko Crevost et Lemarie Ameliorate Loperamide-Induced Constipation in Mice by Regulating Gut Microbiota and Related Metabolites
Source: Int J Mol Sci. 2023 Apr 13;24(8):7191. doi: 10.3390/ijms24087191 (PMC10139007; doi:10.3390/ijms24087191)
Supplement: Supplementary file 1 [file ijms-24-07191-s001.zip › ijms-2308588-supplementary.pdf]

Table S1. Contents of different classifications in ATTF

Classification	Relative abundance (%)
Flavonoids	72.22
Nucleotide	6.52
Phenols	4.30
Alkaloids	3.76
Benzene, Lipids	2.19
Amino acid	1.64
Organic acids, Vitamins	1.17
phytohormone	0.93
Terpene	0.90
Steroids	0.43

Table S2. Chemical compounds of ATTF

Compounds	Class	CAS	Relative abundance (%)
(+)-Epicatechin	Flavonoids	35323-91-2	25.79
(-)-Catechin	Flavonoids	18829-70-4	11.83
L-Epicatechin	Flavonoids	490-46-0	7.44
Isoquercitrin	Flavonoids	482-35-9	7.19
Procyanidin B2	Flavonoids	29106-49-8	5.89
(-)-Epiafzelechin	Flavonoids	24808-04-6	3.85
D-alpha-Aminobutyric acid	Carboxylic acids and derivatives	2623-91-8	2.26
Guanosine	Nucleotide and its derivates	118-00-3	1.99
Adenosine	Nucleotide and its derivates	58-61-7	1.91
Cyanidin-3-rutinoside	Flavonoids	28338-59-2	1.75
Quercetin-3-0-glucuronide	Flavonoids	22688-79-5	1.74
quercetin 3-O-β-D-glucofuranoside	Flavonoids	21637-25-2	1.68
Guanine	Nucleotide and its derivates	73-40-5	1.60
Phyllalbine	Alkaloids	4540-25-4	1.57
Enol-phenylpyruvate	Benzene and substituted derivatives	5801-57-0	1.48
p-Octopamine	Phenols	104-14-3	1.23
2,5-Dihydroxybenzaldehyde	Phenols	1194-98-5	1.19
Riboflavine	Vitamins	83-88-5	1.08
Astragalin	Flavonoids	480-10-4	1.07

Table S3. Primers sequences used for quantitative PCR analysis of gene expression

Primer	Sequence (5' to 3')
RPL-19	GAAGGTCAAAGGGAATGTGTTCA
	CCTTGTCTGCCTTCAGCTTGT
5-HT₂	CATTGCGGGAAACATACTGGT
	CCAGCAGCATATCAGCTATGG
TRPA1	GTCCAGGGCGTTGTCTATCG
	CGTGATGCAGAGGACAGAGAT
PLA2	CTATGCCTTCTATGGATGCCAC
	CAGCCGTTTCTGACAGGAGT
COX2	TGAGCAACTATTCCAAACCAGC
	GCACGTAGTCTTCGATCACTATC
MLC3	ATGGGTGCTGAAATCCGTCAT
	CCGCCACTAGCATCTCTACTTC

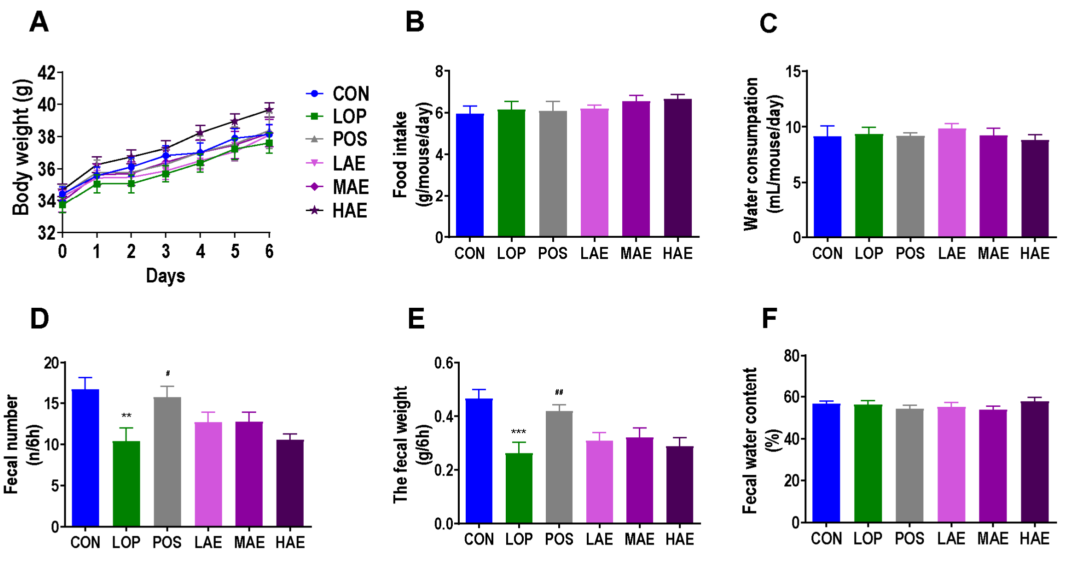

Figure S1. Effects of ATAE on loperamide-induced constipation symptoms in mice. (A)The body weight; (B)Food intake; (C)The water consumption; (D) Number of feces excreted in 6 h, FN; (E) Wet weight of feces excreted in 6 h, FW; (F) Fecal water content. The data are expressed as the means ± SEMs (*n* =10-12). *, compared with the CON group; #, compared with the LOP group. **, *P* < 0.01; ***, *P* < 0.001. #, *P* < 0.05; ##, *P* < 0.01; ###, *P* < 0.001.

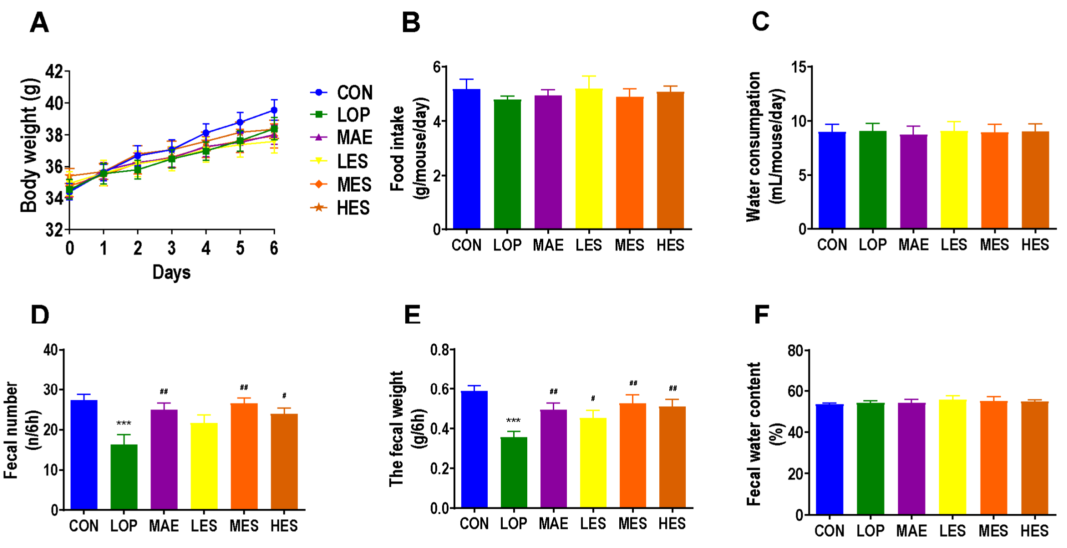

Figure S2. Effects of ATES on loperamide-induced constipation symptoms in mice. (A)The body weight; (B)Food intake; (C)The water consumption; (D) Number of feces excreted in 6 h, FN; (E) Wet weight of feces excreted in 6 h, FW; (F) Fecal water content. The data are expressed as the means ± SEMs (n = 10-12). *, compared with the CON group; #, compared with the LOP group. **, $P < 0.01$; ***, $P < 0.001$. #, $P < 0.05$; ##, $P < 0.01$; ###, $P < 0.001$.

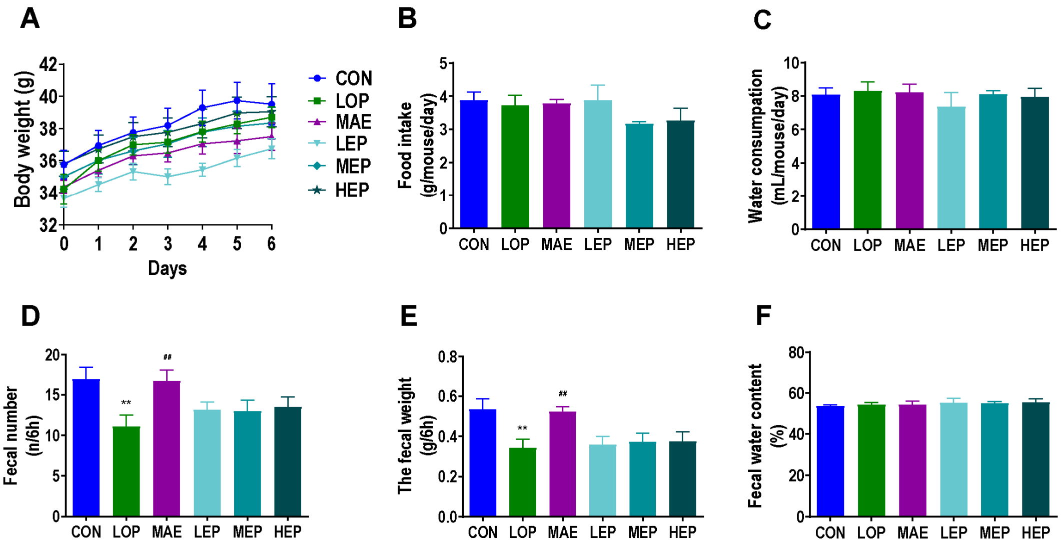

Figure S3. Effects of ATEP on loperamide-induced constipation symptoms in mice. (A)The body weight; (B)Food intake; (C)The water consumption; (D) Number of feces excreted in 6 h, FN; (E) Wet weight of feces excreted in 6 h, FW; (F) Fecal water content. The data are expressed as the means ± SEMs (n = 10-12). *, compared with the CON group; #, compared with the LOP group. **, $P < 0.01$; ***, $P < 0.001$. #, $P < 0.05$; ##, $P < 0.01$; ###, $P < 0.001$.

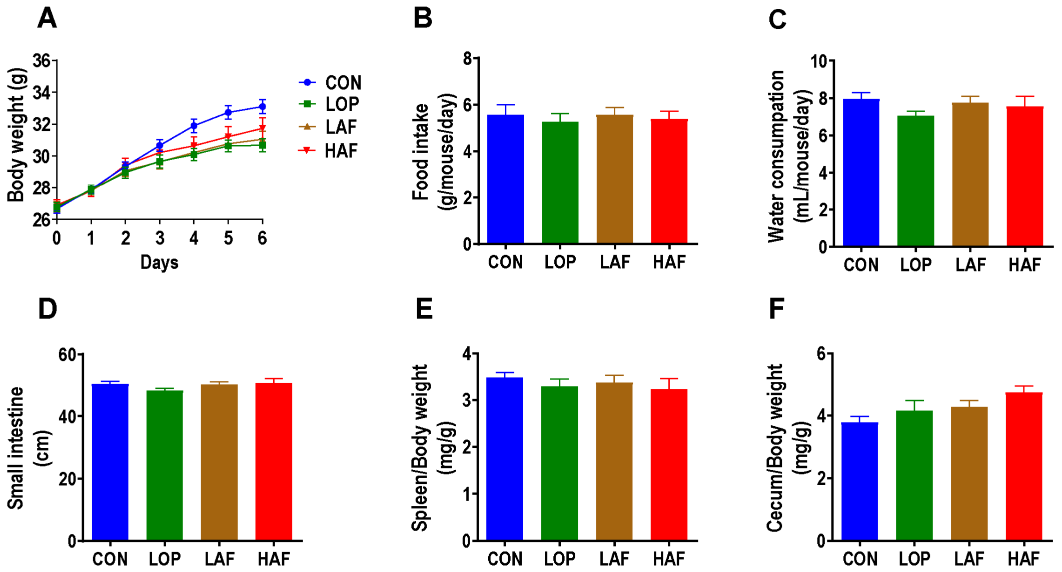

Figure S4. Effects of ATTF on loperamide-induced constipation symptoms in mice. (A)The body weight; (B)Food intake; (C)The water consumption; (D) The small intestine length; (E) Spleen index; (F) Caecum index.

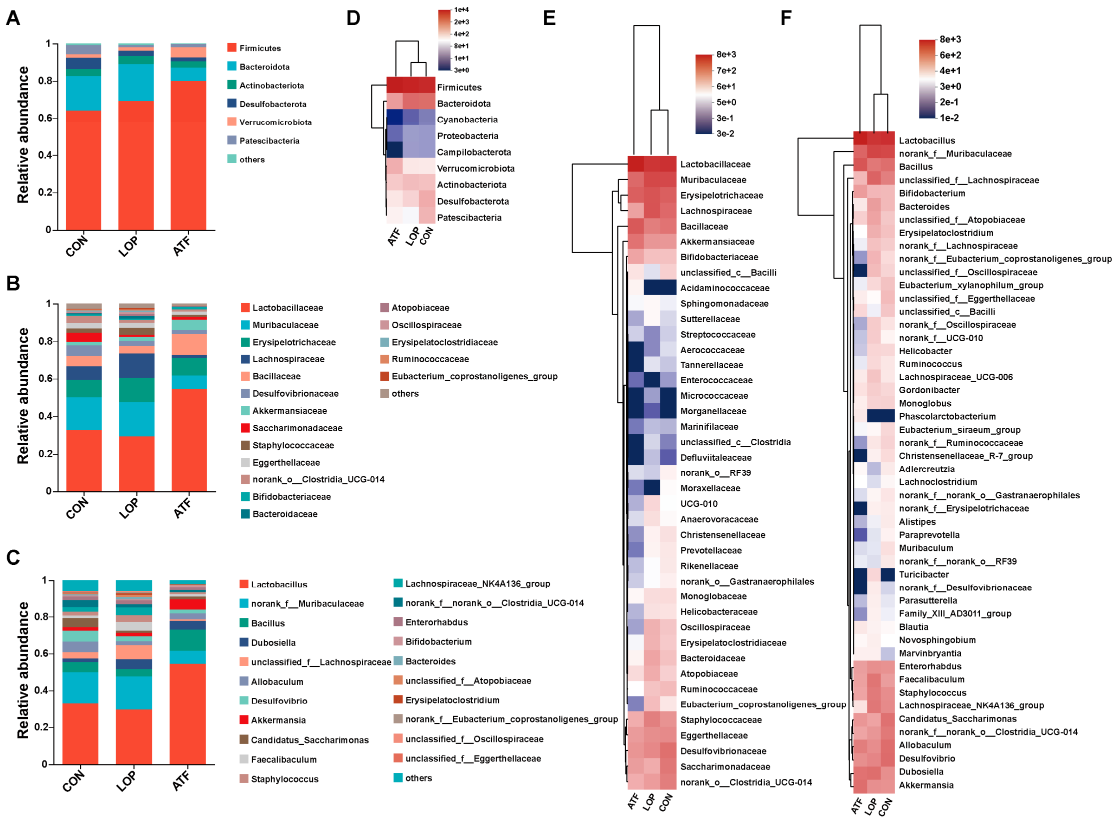

Figure S5. Effect of ATTF on the caecum microbial composition of STC mice. (A) Phylum level. (B) Family level. (C) Genus level. (D to F) Cluster heatmaps of gut microbiota in different groups.

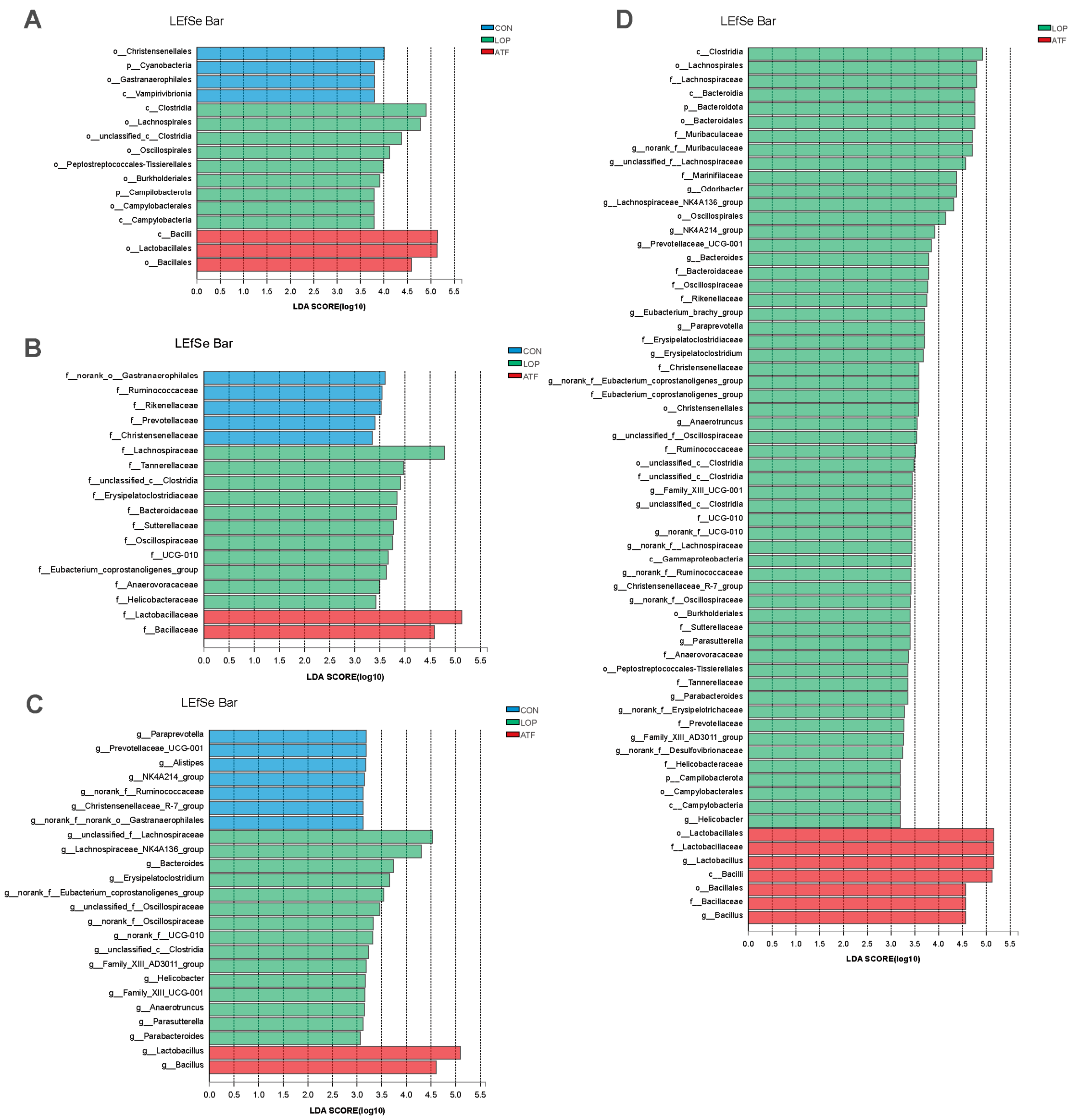

Figure S6. Linear discriminant analysis effect size (LEfSe) analyses (LDA score > 2.0). (A to C) LEfSe analyses based on the CON, LOP and HAF groups. (A) At the phylum, class and order level. (B) At the family level. (C) At the genus level. (D) LEfSe analyses based on the LOP and HAF groups, from the phylum level to the genus level.

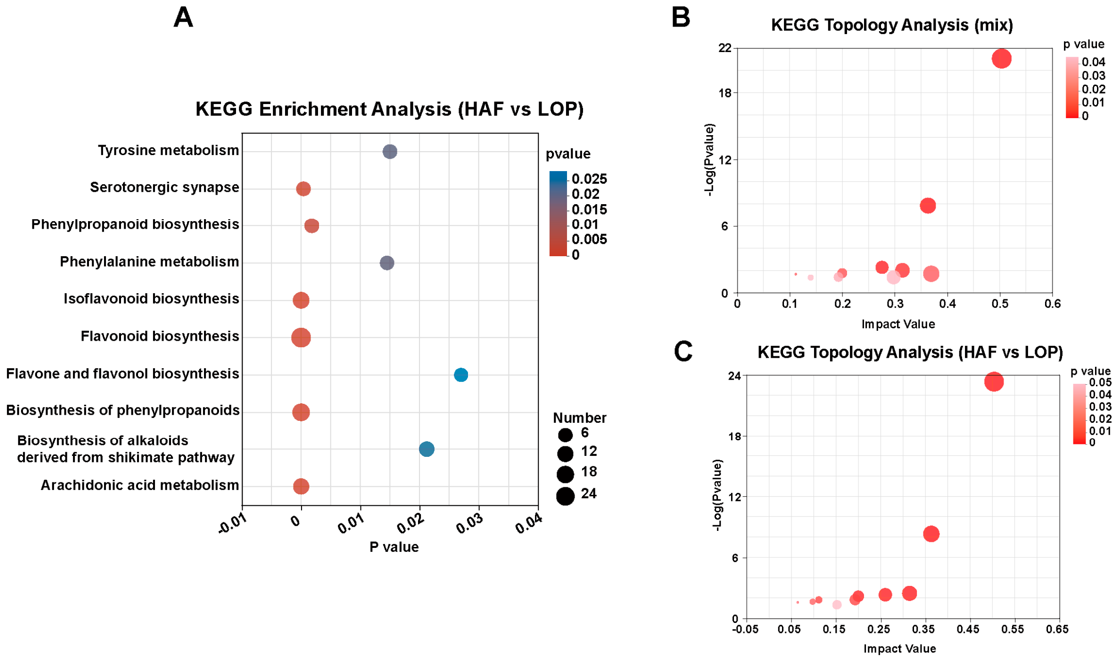

Figure S7. HAF changed the loperamide-induced gut metabolite composition. (A) KEGG topology analysis, (B) KEGG enrichment analysis and (C) KEGG topology analysis based on the LOP and HAF groups. $P < 0.05$, $n = 8$ in each group.

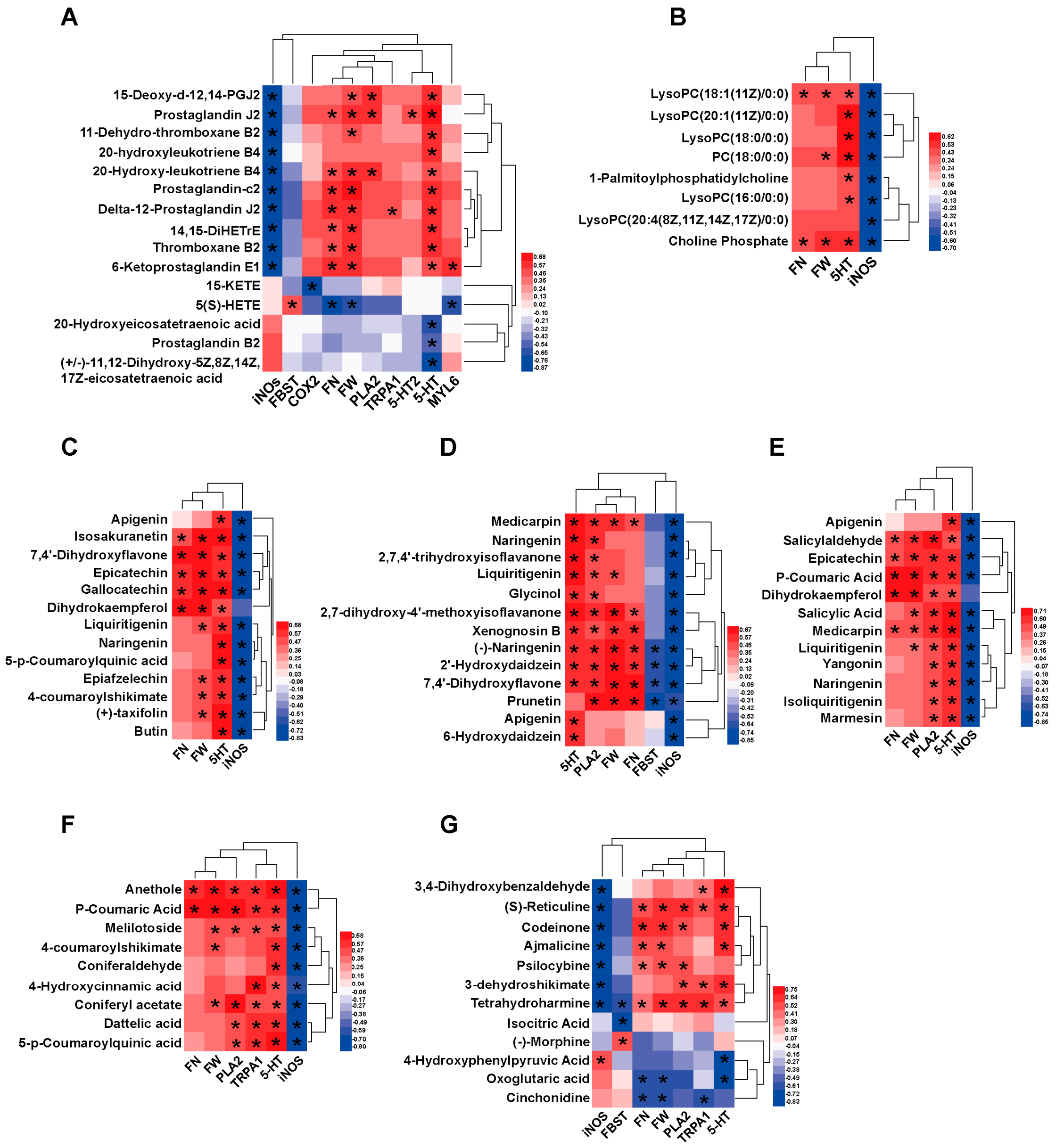

Figure S8. Heatmaps showing bivariate correlations between specific gut metabolites and core host parameters in STC mice. Correlations between differential host parameters and gut metabolites of (A) flavonoid biosynthesis, (B) biosynthesis of phenylpropanoids, (C) isoflavonoid biosynthesis, (D) phenylpropanoid biosynthesis, (E) biosynthesis of alkaloids derived from shikimate pathway, (E) choline metabolism in cancer. The colour at each intersection indicates the value of the *r* coefficient; *P* values were adjusted for multiple testing according to the *BH* procedures. * indicates a significant correlation between these two parameters ($P < 0.05$, $n = 6$ in each group).

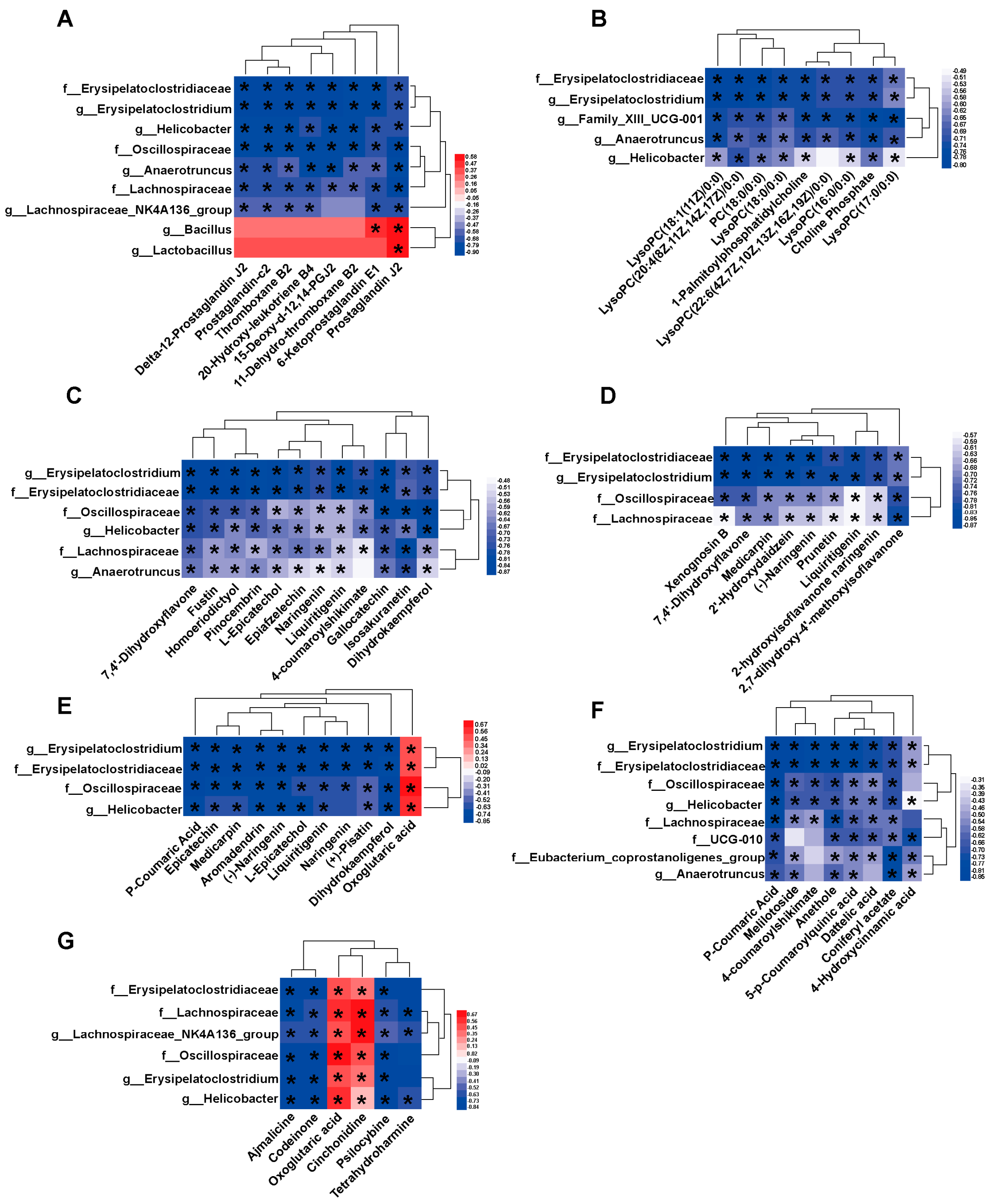

Figure S9. Heatmaps showing bivariate correlations between specific gut microbiota and gut metabolites in STC mice. Correlations between the differential microbe taxa and gut metabolites of (A) flavonoid biosynthesis, (B) biosynthesis of phenylpropanoids, (C) isoflavonoid biosynthesis, (D) biosynthesis of alkaloids derived from shikimate pathway, (E) phenylpropanoid biosynthesis, (E) choline metabolism in cancer. The colour at each intersection indicates the value of the *r* coefficient; *P* values were adjusted for multiple testing according to the *BH* procedures. * indicates a significant correlation between these two parameters ($P < 0.05$, $n = 6$ in each group).
